# Immediate and Long-Term Adverse Events of COVID-19 Vaccines: A One-Year Follow-Up Study From the Kurdistan Region of Iraq

**DOI:** 10.7759/cureus.47670

**Published:** 2023-10-25

**Authors:** Manhal A Abdulkader, Muayad A Merza

**Affiliations:** 1 Department of Clinical Pharmacy, College of Pharmacy, University of Duhok, Duhok, IRQ; 2 Department of Internal Medicine, University of Duhok, Duhok, IRQ

**Keywords:** kurdistan region of iraq, sars-cov-2, long term, short term, prevention, sinopharm, astrazeneca, pfizer, adverse event, covid-19 vaccine

## Abstract

Background

The administration of COVID-19 vaccines has been critical in controlling the spread of the virus. However, understanding the potential adverse events (AEs) associated with these vaccines is crucial for public health. While most previous studies observed only short-term AEs, this study aimed to investigate the immediate and long-term AEs following the first and second doses of Pfizer, AstraZeneca, and Sinopharm vaccines, providing valuable long-term insights.

Methodology

A prospective, one-year, follow-up study was conducted by tracking 922 vaccinated individuals to assess short-term and long-term AEs. Demographics, clinical characteristics, vaccine types, and dose effects were taken into consideration. AEs were classified based on severity and duration. Statistical analyses were performed to compare differences among the vaccine groups, with p-values <0.05 considered significant. Bowker’s and chi-square tests were performed using JMP Pro 14.3.0.

Results

Of the 922 participants, 55.53% (n = 512) were vaccinated with Pfizer, and 23.32% (n = 215) and 21.15% (n = 195) were vaccinated with Sinopharm and AstraZeneca, respectively. Overall, 72.34% of participants (n = 667) were suffering from AEs after the first dose, with a lower prevalence of AEs after the second dose (52.71%, n = 486). Pfizer exhibited the highest percentage and severity of AEs, followed by AstraZeneca and Sinopharm. Most AEs reported in this study were mild and resolved within 72 hours, with females experiencing more frequent AEs. The common short-term AEs observed were fever, injection-site pain, myalgia, fatigue, and headache. Notably, there were no chronic AEs, and only one case of myocarditis was associated with AstraZeneca.

Conclusions

Despite the variation in the prevalence of AEs among the three vaccines, the vaccination process proved to be safe with no serious short-term AEs. However, the long-term AEs associated with AstraZeneca and the decrease in the prevalence of AEs after the second dose of the COVID-19 vaccines warrant further investigations and priority for future research.

## Introduction

The COVID-19 pandemic has dramatically impacted the world since it first emerged in Wuhan, China, in late 2019 [1]. The importance of developing effective strategies to manage the pandemic and prevent the spread of the virus cannot be overstated. One key approach to mitigating the risk of COVID-19 is through vaccination. The COVID-19 vaccines have been developed and authorized for emergency use in record time, with promising results in clinical trials. Three vaccines, namely, Pfizer-BioNTech, Sinopharm, and AstraZeneca, were the first to receive emergency authorization for use against COVID-19 [2]. Two doses of the vaccines are required for maximum efficacy. These vaccines were introduced nationally in Duhok city starting with the first vaccination dose of Sinopharm on March 03, 2021, followed by AstraZeneca and Pfizer at the beginning and middle of April 2021, respectively [3].

Based on the literature, most reported adverse events (AEs) are considered mild to moderate, including local and systemic reactions such as pain, swelling, redness at the injection site, fever, headache, fatigue, and muscle aches [4-6]. However, clinical trials for COVID-19 vaccines have been conducted in controlled settings, with specific inclusion and exclusion criteria, which may not fully represent the general population as real-world research [7]. Real-world research can also help identify any rare AEs that may not have been detected in clinical trials due to their small sample sizes or limited follow-up duration. Therefore, this study aims to provide actual evidence about AEs experienced by vaccine recipients and compare them among the three vaccines in terms of safety and tolerance. Of note, this is the first prospective longitudinal study in Iraq (and probably the first in the Middle East unless worldwide) designed to follow vaccinated individuals for one year targeting long-term AEs associated with COVID-19 vaccination.

## Materials and methods

A prospective (longitudinal) study was conducted among adult individuals who were eligible for vaccination. The study was conducted at vaccination centers in Duhok City in the Kurdistan region, Iraq. Data collection commenced immediately following the approval of the research protocol by the ethics committee, as indicated in the official letter issued by the Department of Health in Duhok (reference number: 241020201-10-2). Eligible participants for this study were individuals aged 18 years and above who had received the complete regimen of either the Sinopharm, Pfizer-BioNTech, or AstraZeneca COVID-19 vaccine. Individuals were considered eligible if they had received or were willing to receive two doses of the COVID-19 vaccine (fully vaccinated) of any vaccine type available in Duhok City during the study period. The inclusion criteria included (1) individuals 18 years old and above eligible for vaccination; (2) had to receive the first dose of the vaccine at the time of data collection; (3) willing to continue with the follow-up program of the study after receiving the second dose of vaccine. Exclusion criteria encompassed individuals who had received only one dose of the aforementioned vaccines, those who did not receive the second dose within the recommended interval, or individuals with current or past COVID-19 infection in the previous six months (because the immune response of such patients is different from infection-naive ones and may affect the AEs of the vaccine). Any loss of follow-up led to the exclusion of the case from the study. An individual was considered partially vaccinated 21 days after receiving the first vaccine and fully vaccinated 14 days after receiving the second dose of the vaccine [8]. The sample was collected randomly using the following formula for sample size calculation: n = z^2^ × p × (1 - p)/SE^2^. In this study, we used a sample size of n, a z-score of 1.96 associated with a 95% confidence level, a sample proportion of 0.5 expressed as a decimal, and a margin of error of 0.05 expressed as a decimal. We chose p = 0.5 as it is the most conservative value that maximizes the sample size. We also used SE of 0.05, which means we aimed for a margin of error of 5%. Plugging these values into the formula, we get n = 1.96^2^ × 0.5 × (0.5)/0.05^2^, which led to 384.16, which was rounded up to 385 as the sample size needed. However, we achieved a total of 992 vaccinated individuals.

Data collection tool

Based on direct face-to-face interviews, data were collected using a well-designed data collection form. The data collection form was reviewed for validity by three specialists, a clinical pharmacist, a medical specialist of community health, and a professor of infectious diseases, who were familiar with and had expertise in vaccination programs. The validity was checked for generalizability, fluency, the time required to answer and fill the form, and concordance with the literature and content. Data collection started after obtaining study approval. Based on direct face-to-face interviews, data were collected using a well-designed questionnaire (Appendices). The questionnaire was reviewed for validity by a panel of three specialists, a clinical pharmacist, a medical specialist of community health, and a professor of infectious diseases, who were familiar with and had expertise in vaccination programs. A pilot study was done among 20 participants considering their feedback to refine the data collection form. Their responses were not included in the final data set for analysis. The first part of the questionnaire included the participant’s name, contact details, and consent form for agreement to participate in the study. The second part included sociodemographic data and medical characteristics. The medical part involved questions on chronic diseases, such as hypertension and diabetes, and any other disease, such as immunodeficiency or autoimmune diseases. Daily medication use was also documented. The third part included vaccine information and the AEs that occurred within the first few days after vaccination (from the first day till day eight). The severity of AEs was also documented and classified into mild, moderate, and severe according to the intensity of the AEs. This was based on two severity rating categories of AEs, the first one derived from a Likert-based rating system that ranged from none (no symptoms) to severe (prevented regular daily activity). The second matched the severity level of vaccine-related AEs classified into three categories based on their severity score, i.e., mild (1-3), moderate (4-7), and severe (8-10). By combining these two categorization methods, we create a comprehensive table that can be used to assess the severity of vaccine-related AEs in this study. The number of AEs each individual suffered from was also reported and classified into none, one to three AEs, and more than three AEs based on how many AEs the vaccinated suffered from after each vaccine shot. The body mass index (BMI) was calculated based on self-reported body weight and height and then categorized into five groups, i.e., underweight (<18.50 kg/m²), normal weight (18.50-24.99 kg/m²), overweight (25.00-29.90 kg/m²), obesity (30.00-39.90 kg/m²), and severe obesity (>40.00 kg/m²). The fourth part is related to the follow-up process. Age was classified according to the WHO criteria for human life [9] into young age (18-43 years old), middle age (44-59 years old), old age (60-74 years old), and senile age (75-90). Additionally, we performed a long-term active follow-up for the vaccinated individuals (weekly) to detect long-term AEs and vaccine-related complications, which is an additional aim of this study in addition to the evaluation of short-term AEs. The long-term AE is any AE, reaction, or complication that may occur for a vaccinated individual after day eight of vaccination with no apparent reason other than the vaccine.

Statistical analysis

Statistical calculations were performed using JMP Pro 14.3.0. (https://www.jmp.com/en_us/home.html). Demographic and medical characteristics were expressed as numbers and percentages. The AEs experienced post-vaccination were presented as a percentage, and the difference between partially and fully vaccinated individuals was tested using Bowker’s test (equivalent to the McNemar test). The difference in AEs among the types of vaccines based on doses was also performed using Pearson’s chi-square test. The significant level of deference was set at p-values <0.05.

## Results

Demographics and clinical characteristics

Of the 922 participants enrolled in the study, 484 (52.6%) were females. Approximately 564 (61.17%) participants were aged 18 to 43 years. Almost half of the participants were overweight, with a mean BMI of the entire study population of 26 kg/m^2^ (SD = 3.52 kg/m^2^). More than half of the study population were vaccinated with Pfizer (512, 55.53%). The number of individuals vaccinated with Sinopharm and AstraZeneca was 215 (23.32%) and 195 (21.15%), respectively. The demographics and clinical characteristics of the study population are presented in Table 1.

**Table 1:** Demographic and clinical characteristics of COVID-19 vaccine receivers. BMI = body mass index; SEM = standard error of the mean

General characteristics (n = 922)	Statistics	
	Number	Percentage (%)
Gender		
Male	437	47.40
Female	487	52.60
Age (18–86 years)/SEM = 0.45	Mean = 39.77	SD = 13.76
Age group		
18–43	564	61.17
44–59	272	29.50
60–74	81	8.79
75–89	5	0.54
BMI (range = 15.24-45.78 kg/m^2^)	Mean = 26.00	SD = 3.52
BMI category		
Underweight <18.5	10	1.09
Normal weight 18.5–24.90	314	34.06
Overweight (25.0 ≤ BMI < 29.90)	435	47.18
Obese (30.0 ≤ BMI ≤ 39.90)	155	16.81
Severe obesity (BMI>40.0)	8	0.87
Level of education		
None	155	16.81
Primary	158	17.14
Secondary	223	24.19
University	266	28.85
Postgraduate	120	13.05
Smoking status (cigarette)		
No	723	78.42
Yes	199	21.58
Alcohol		
No	906	98.27
Yes	16	1.74
Hookah		
No	873	94.69
Yes	49	5.32
Pregnant		
No	916	99.35
Yes	6	0.65
Blood group		
A	309	33.51
B	146	15.84
O	400	43.38
AB	67	7.27
Chronic diseases		
No	700	75.92
Yes	222	24.08
On medication		
No	732	79.40
Yes	190	20.60
Vaccine type		
Pfizer	512	55.53
Sinopharm	215	23.32
AstraZeneca	196	21.15

**Figure 1:**
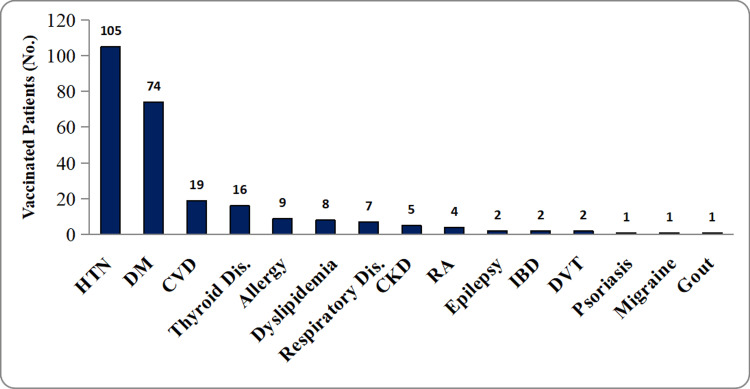
Types of comorbidities among the study population. HTN = hypertension; DM = diabetes mellitus; CVD = cardiovascular diseases; CKD = chronic kidney disease; RA = rheumatoid arthritis; IBD = irritable bowel disease; DVT = deep venous thrombosis; Dis = disease

Approximately one-quarter of the study population (222, 24.08%) had at least one chronic disease, and an additional 47 (5.1%) had multiple diseases. Hypertension was the most commonly observed comorbidity (Figure 1). Other common comorbidities included diabetes mellitus, cardiovascular diseases, chronic kidney diseases, and thyroid disorders. Other less common diseases are presented in Figure 1. Accordingly, 20.6% (n = 190) of the participants were on regular use of medications. The most commonly used medications were anti-hypertensive, anti-diabetes, and anti-platelet medications.

Adverse events following the first and second doses of COVID-19 vaccination

The first comparison between the AEs in this study was made for the first dose receivers with second dose receivers which included the three types of vaccines. The majority of the study population (72.34%, n = 667) experienced at least one AE after the first dose, with approximately only half of the study participants (52.71%, n = 486) having AEs after the second dose of the vaccine. This difference in AE prevalence between the two doses was significant (p < 0.0001). The most frequent AEs after both the first and second vaccine doses were injection-site pain, fever, myalgia, headache, fatigue, and chills (Table 2).

**Table 2:** AEs of COVID-19 vaccine after the first and second doses. Bowker’s test (equivalent to the McNemar test) was performed for statistical analyses. AEs = adverse events

Medical characteristics (n = 922)	Frequency of AEs, N (%)		P-value
	First dose	Second dose	
Signs and symptoms (AEs)			
Asymptomatic	255 (27.66)	436 (47.29)	<0.0001
Symptomatic	667 (72.34)	486 (52.71)	
Fever			
No	659 (71.48)	728 (78.96)	<0.0001
Yes	263 (28.52)	194 (21.04)	
Fatigue			
No	689 (74.73)	822 (89.15)	<0.0001
Yes	233 (25.27)	100 (10.85)	
Headache			
No	693 (75.16)	767 (b3.38)	<0.0001
Yes	229 (24.84)	154 (16.72)	
Myalgia			
No	670 (72.67)	919 (88.83)	<0.0001
Yes	252 (27.33)	103 (11.17)	
Chills			
No	882 (95.66)	906 (98.27)	0.0007
Yes	40 (4.34)	16 (1.73)	
Nausea			
No	913 (99.02)	916 (99.35)	0.4053
Yes	9 (0.98)	6 (0.65)	
Cough			
No	909 (98.59)	917 (99.47)	0.0290
Yes	13 (1.41)	5 (0.54)	
Shortness of Breath			
No	921 (99.89)	921 (99.89)	1.000
Yes	1 (0.11)	1 (0.11)	
Loss of taste and smell			
No	921	918 (99.57)	0.1797
Yes	1 (0.11)	4 (0.43)	
Injection-site pain			
No	661 (71.69)	663 (71.91)	0.8208
Yes	261 (28.31)	259 (28.09)	
Duration of AEs			
None	255 (27.66)	436 (47.29)	<0.0001
Less than 24 hours	109 (11.82)	148 (16.05)	
24–48 hours	504 (54.66)	284 (30.80)	
More than 24 hours	54 (5.86)	54 (5.86)	
Number of AEs			
None	255 (27.66)	436 (47.29)	<0.0001
1–3	604 (65.51)	468 (50.76)	
>3	63 (6.83)	18 (1.95)	
Mean of AEs	1.37 (1.24) 0–6	1.79 (0.85) 1–5	
Severity of AEs			
No AEs	255 (27.66)	436 (47.29)	0.0034
Mild	469 (50.87)	296 (32.10)	
Moderate	117 (12.69)	113 (12.26)	
Severe	81 (8.79)	77 (8.35)	

Distribution of adverse events among the three types of vaccines

When comparing the frequency of AEs among the three vaccine types, we found that the inactivated Sinopharm vaccine receivers had the lowest percentage of AEs compared to both Pfizer and AstraZeneca vaccine receivers (Table 3). The severity was classified into mild, moderate, and severe depending on the intensity of symptoms experienced by the vaccinated individual and on the need for management, as well as on the setting and type of management applied. Occasionally, for all three vaccine types in both doses, the majority of vaccinated individuals had mild-to-moderate levels of severity. Notably, Pfizer was associated with the highest number of severe AEs (13.78, n = 71) compared to Sinopharm (3.7%, n = 8) and AstraZeneca (1.02%, n = 2) after the first and second doses. For both, the first and second doses majority of symptomatic vaccinated subjects had one to three AEs with the duration of AEs between 24 and 48 hours for the three vaccines, as shown in Table 3. Nevertheless, the incidence of AEs exceeding three in number was notably higher among recipients of the Pfizer vaccine. Further, Pfizer vaccine recipients exhibited the highest percentage of AEs persisting for over 48 hours, relative to the other vaccines, following both the first and second doses.

**Table 3:** Comparisons of AE rates among different vaccine types for vaccinated individuals after the first and second doses. Pearson’s chi-square tests were performed for statistical analyses. AE = adverse event

AE type	First dose				Second dose			
	Vaccine type, N (%)			P-value (two-sided)	Vaccine type, N (%)			P-vale (two-sided)
	Pfizer	SinoPharm	AstraZeneca		Pfizer	SinoPharm	AstraZeneca	
Fever								
No	345 (67.38)	189 (87.91)	125 (64.10)	<0.0001	382 (74.61)	199 (92.56)	147 (75.38)	<0.0001
Yes	167 (32.62)	26 (12.09)	70 (35.90)		130 (25.39)	16 (7.44)	48 (24.62)	
Fatigue								
No	351 (68.55)	182 (84.65)	156 (80.00	<0.0001	470 (91.80)	181 (84.19)	171 (87.69)	0.0082
Yes	161 (31.45)	33 (15.35)	39 (20.00)		42 (8.20)	34 (15.81)	24 (12.31)	
Headache								
No	375 (73.24)	193 (89.77)	125 (64.10)	<0.0001	423 (82.78)	187 (86.98)	157 (80.51)	0.1945
Yes	137 (26.76)	22 (10.23)	70 (35.90)		88 (17.22)	28 (13.02)	38 (19.49)	
Myalgia								
No	333 (65.04)	202 (93.95)	135 (69.23)	<0.0001	447 (87.30)	203 (94.42)	169 (86.67)	0.0118
Yes	179 (34.96)	13 (6.05)	60 (30.77)		65 (12.70)	12 (5.58)	26 (13.33)	
Injection-site pain								
No	371 (72.46)	160 (74.42)	130 (66.67)	0.1861	337 (65.82)	164 (76.28)	162 (83.08)	<0.0001
Yes	141 (27.54)	55 (25.58)	65 (33.33)		175 (34.18)	51 (23.72)	33 (16.92)	
Chills								
No	490 (95.70)	201 (93.49)	191 (97.95)	0.0860	504 (98.44)	211 (98.14)	191 (97.95)	0.8942
Yes	22 (4.30)	14 (6.51)	4 (2.05)		8 (1.56)	4 (1.86)	4 (2.05)	
Nausea								
No	508 (99.22)	212 (98.60)	193 (98.97)	0.4719	506 (98.83)	215 (100)	195 (100)	0.0891
Yes	4 (0.78)	3 (1.40)	2 (1.03)		6 (1.17)	0 (0.00)	0 (0.00)	
Cough								
No	502 (98.05)	214 (99.53)	193 (98.97)	0.2626	509 (99.41)	213 (99.07)	195 (100)	0.4314
Yes	10 (1.95)	1 (0.47)	2 (1.03)		3 (0.59)	2 (0.93)	0 (0.00)	
Loss of taste and smell								
No	511 (99.80)	215 (100)	195 (100)	0.6698	509 (99.41)	214 (99.53)	195 (100)	0.5687
Yes	1 (0.20)	0 (0.00)	0 (0.00)		3 (0.59)	1 (0.47)	0 (0.00)	
Shortness of breath								
No	512 (100)	214 (99.53)	195 (100)	0.1928	511 (99.80)	215 (100)	195 (100)	0.6698
Yes	0 (0.00)	1 (0.47)	0 (0.00)		1 (0.20)	0 (0.00)	0 (0.00)	
AEs								
No	88 (17.19)	134 (62.3)	33 (16.9)	<0.0001	211 (41.21)	133 (61.86)	92 (47.18)	<0.0001
Yes	424 (82.81)	81 (37.7)	162 (83.1)		301 (58.79)	82 (38.14)	103 (52.82)	
Severity of AEs								
None	88 (17.19)	134 (62.3)	33 (16.92)	<0.0001	211 (41.21)	133 (61.86)	92 (47.18)	<0.0001
Mild	263 (51.37)	57 (26.5)	149 (76.41)		179 (34.96)	51 (23.72)	65 (33.33)	
Moderate	90 (17.58)	16 (7.4)	11 (5.64)		63 (12.3)	24 (11.16)	27 (13.85)	
Severe	71 (13.87)	8 (3.7)	2 (1.03)		59 (11.52)	7 (3.26)	11 (5.64)	
Number of AEs								
None	88 (17.19)	134 (62.3)	33 (16.9)	<0.0001	211 (41.21)	133 (61.86)	92 (47.18)	<0.0001
1–3	383 (74.80)	65 (30.2)	157 (80.5)		287 (56.05)	79 (36.74)	102 (52.31)	
>3	41 (8.01)	16 (7.4)	5 (2.6)		14 (2.73)	3 (1.4)	1 (0.51)	
Duration of AEs								
None	88 (17.19)	134 (62.3	33 (16.9)	<0.0001	211 (41.21)	133 (61.86)	92 (47.18)	<0.0001
<24 hours	57 (11.13)	22 (10.2)	30 (15.4)		57 (11.13)	39 (18.14)	52 (26.67)	
24–48 hours	322 (62.89)	55 (25.6)	127 (65.1)		196 (38.28)	39 (18.14)	49 (25.13)	
>48 hours	45 (8.79)	4 (1.9)	5 (2.6)		48 (9.38)	4 (1.86)	2 (1.03)	

Number of adverse events with demographic and clinical characteristics

The number of AEs was classified into three categories (none 1-3, and more than three AEs) to facilitate the comparison. The percentages of females versus males having one to three AEs after the first and second dose were 69.38% (n = 336) versus 60.64% (n = 265) and 54.64% (n = 265) versus 46.45% (n = 203), respectively. For multiple AEs category, females had a higher percentage than males after both the first and second doses with more than double that of males (9.17%, n = 44 versus 4.35%, n 19) after the first dose and even higher variation after the second dose with a statistical difference, as shown in Table 4.

**Table 4:** Comparisons of the number of AEs for vaccinated individuals with demographic and clinical characteristics. Pearson’s chi-square tests were performed for statistical analyses. AE = adverse event; BMI = body mass index

Subject characteristics (n = 922)	Number of AEs after the first dose, N (%)				Number of AEs after the second dose, N (%)			
	None	1–3	>3	P-value (two-sided)	None	1–3	>3	P-value (two-sided)
Gender								
Male	153 (35.01)	265 (60.64)	19 (4.35)	<0.0001	231 (52.86)	203 (46.45)	3 (0.69)	0.0005
Female	105 (21.65)	336 (69.38)	44 (9.17)		205 (42.27)	265 (54.64)	15 (3.09)	
Age group (years)								
18–43	155 (27.58)	368 (65.25)	41 (7.37)	0.800	259 (45.92)	297 (52.66)	8 (1.42)	0.2013
44–59	77 (28.31)	178 (65.44)	17 (6.35)		128 (47.06)	135 (49.63)	9 (3.31)	
60–74	23 (28.40)	53 (65.43)	5 (6.27)		45 (55.56)	35 (43.21)	1 (1.23)	
75–89	3 (60.00)	2 (40.00)	0 (0.00)		4 (80.00)	1 (20.00)	0 (0.00)	
BMI category								
Underweight	3 (30.00)	6 (60.00)	1 (10.00)	0.400	6 (60.00)	3 (30.00)	1 (10.00)	0.0300
Normal weight	100 (31.85)	198 (62.42)	18 (5.73)		159 (50.64)	152 (48.41)	3 (0.96)	
Overweight	106 (24.47)	296 (68.05)	33 (7.69)		186 (42.76)	240 (55.17)	9 (2.07)	
Obese	48 (31.07)	96 (61.94)	11 (7.10)		83 (53.55)	67 (43.23)	5 (3.23)	
Severe obesity	1 (12.50)	7 (87.50)	0 (0.00)		2 (25.00)	6 (75.00)	0 (0.00)	
Smoking status								
No	192 (26.56)	477 (65.98)	54 (7.5)	0.1571	326 (45.09)	382 (52.84)	15 (2.07)	0.0383
Yes	63 (31.66)	127 (63.82)	9 (4.5)		110 (55.28)	86 (43.22)	3 (1.51)	
Alcohol								
No	251 (27.73)	591 (65.30)	63 (6.96)	0.2577	425 (46.91)	463 (51.10)	18 (1.99)	0.2096
Yes	7 (43.75)	9 (56.25)	0 (0.00)		11 (68.75)	5 (31.25)	0 (0.00)	
Hookah								
No	243 (27.84)	571 (65.41)	59 (6.76)	0.8243	411 (47.08)	444 (50.86)	18 (2.06)	0.5502
Yes	15 (30.61)	30 (61.22)	4 (8.16)		25 (51.02)	24 (48.98)	0 (0.00)	

## Discussion

AEs associated with COVID-19 vaccines have been a topic of significant interest and concern. When comparing the AEs of Pfizer, Sinopharm, and AstraZeneca vaccines, it is important to consider factors such as vaccine technology, composition, and population demographics, as these can influence the occurrence and severity of AEs. According to our study results, females had more AEs than males, and the difference was statistically significant after the first (p < 0.0001) and second doses (p < 0.0005). The percentage of symptomatic individuals for males versus females was 65.67% versus 78.35% and 47.14% versus 57.73% after the first and second doses, respectively (p < 0.0001). In addition to the observed gender disparity related to the frequency of AEs in our study, the percentage of severe AEs among females was more than double that of males. This finding was in line with another study from Iraq [10] and other countries [11,12]. However, this observation is not limited to COVID-19 vaccines but has been observed across various vaccine types earlier [13,14]. Despite the lack of a full explanation about the higher risk of AEs in females compared to males, hormonal, immunological, and psychological factors could be the main causes behind this disparity. Androgen suppression for immune response renders males with less reactogenicity to vaccines and fewer AEs compared to females [15]. Females also possess a stronger immediate response to the antigen [16] and a lower threshold for coping with pain which is considered an important psychological factor for such disparity in AEs [17].

According to our study results, 667 (72.34%) and 486 (52.7%) study participants were symptomatic after the first and second doses, respectively. Almufty et al. reported a higher proportion of symptomatic individuals (84%) in a retrospective study conducted in Duhok, Kurdistan Region, Iraq. In that study, 36.6% (n = 370) of participants had been infected with COVID-19, which could be a cause for this higher rate compared to our prospective study [18]. Unlike the study by Almufty et al., a previous COVID-19 infection within six months was an exclusion criterion in our study. Another online, retrospective, cross-sectional study in Mosul, which is the nearest city to Duhok, reported a 65% (n = 116) and 77% (n = 512) prevalence of AEs among male and female participants, respectively, after receiving any of the three types of vaccines, i.e., Pfizer, Sinopharm, or AstraZeneca [10]. Despite the lack of similar studies at the national and global level with a methodology that compares the three vaccines at once after both the first and second doses simultaneously, evidence could be taken from nearby countries and worldwide comparable studies considering two vaccines or either after the first or second dose merely. In one online cross-sectional survey conducted to explore the AEs on residents of the UAE, 64.8% (n = 1,217) of study participants reported one or more side effects after at least one dose of Pfizer or Sinopharm vaccines [19].

An unenlightened finding in the literature of COVID-19 AE studies which has been noticed in our study is the lower incidence of AEs after the second dose compared to the first dose, which is contrary to some studies in the literature that reported a higher percentage of AEs after the second dose of several COVID-19 vaccinations [20,21]. In the study by Sultana et al., it was observed that the incidence of AEs after the first dose of mRNA vaccines was the highest for Pfizer (75%) and Moderna (65.04%). However, after the second dose, the percentage of AEs for Pfizer declined to 71.19%, whereas for Moderna, it was 86.09%. It is worth noting that while Pfizer and Moderna vaccines exhibited varying trends in AEs after the second dose, other vaccines such as Sinopharm, Sinovac, and Oxford-AstraZeneca demonstrated a significant reduction in AEs after the second dose during overall immunization [22]. For the AstraZeneca vaccine, this unusual trend of lower AEs after the second dose was also reported in two separate studies from the Kingdom of Saudi Arabia and the United Kingdom with large sample sizes [23,24]. The same decline in the prevalence of AEs was noticed with the Sinopharm vaccine among vaccinated individuals in studies from Iran and the UAE. [19,25]. Another cross-sectional study was conducted in Jordan with participants who had been vaccinated with Pfizer, Sinopharm, or AstraZeneca [26]. The study revealed that after the first dose, 71.8% (n = 1,279) of participants reported experiencing AEs, while the percentage of AEs among participants vaccinated with the second dose was 64.7% (n = 279). These results align closely with our study findings and, when combined with the aforementioned previous studies, provide substantial support for the hypothesis of the decline in AEs following the administration of the second dose.

Despite the considerable number of local and international studies reporting the decline of AEs after the second dose of COVID-19 vaccines, it is important to note that none of these studies have provided a rational discussion or sufficient explanation for this observed trend. Possibly, the cause is related to the immune response modulation effects. It is possible that the initial dose of the vaccine primes the immune system, while the second dose enhances and fine-tunes the immune response. This modulation of the immune response may contribute to a reduced likelihood of AEs after the second dose. Another proposed cause could be the gradual adaptation process. The body may gradually adapt to the vaccine components after the initial dose, leading to decreased reactogenicity upon subsequent doses. Consequently, further research is required to thoroughly investigate and validate the factors contributing to this observed decline in AEs following the administration of the second dose of COVID-19 vaccines knowing that a reduction in the severity of AEs could occur as well [24].

There was no life-threatening or uncommon AE reported post-COVID-19 vaccination among the study population. The most common AEs after the first dose in decreasing order were fever (28.52%), injection-site pain (28.31%), myalgia (27.33%), fatigue (25.27%), and headache (24.84%). After the second dose, they were injection-site pain (28.09%), fever (21.04%), headache (16.72), myalgia (11.17%), and fatigue (10.85%). For all these symptoms (except for injection-site pain), there was a significant difference between the first and second doses, with a decline in the prevalence of these AEs after the second dose, as mentioned earlier (p < 0.0001). Other reported AEs such as nausea, cough, shortness of breath, and loss of taste and smell were rare. Our finding is similar to other study findings locally and worldwide with simple differences in the sequence and frequency of AEs that were reported [10,18,19,23].

After considering each vaccine separately, the percentages of AEs after the first dose for Pfizer and AstraZeneca were very near (82.81% versus 83.1%, respectively), and both were higher than that of Sinopharm. The same trend was observed after the second dose of vaccination, with Pfizer exhibiting the highest percentage of AEs (Table 3). Pfizer also was associated with more severe AEs compared to AstraZeneca and Sinopharm, with Sinopharm exhibiting the lowest severity of AEs. The percentage of vaccinated individuals with more than three AEs was also the highest with Pfizer compared to Sinopharm and AstraZeneca-vaccinated individuals. This difference in the percentage, severity, and number of AEs between the three vaccines was significant (p < 0.0001). The reported higher risk of AEs with Pfizer to be followed by AstraZeneca and then the lowest with Sinopharm was also observed in one systematic review and meta-analysis of 19 studies, Five of these studies were on mRNA vaccines, six on inactivated vaccines, and three on vector vaccines. The analysis demonstrated a substantially higher risk of adverse reactions for the mRNA vaccine with a pooled relative risk (RR) of 2.01 (95% confidence interval (CI) 1.82-2.23), followed by an RR of 1.65 (95% CI = 1.31-2.32) and 1.46 (95% CI = 1.19-1.78) for vector and inactivated vaccines, respectively [27]. These findings shed light on the considerable impact of mRNA vaccines on AEs within the studied populations, highlighting the need for further investigation and monitoring of vaccine safety. Nevertheless, this mRNA technology is relatively new compared to traditional vaccine platforms, which may contribute to differences in side effect profiles. Additionally, mRNA vaccines are designed to stimulate a potent immune response, particularly through the production of spike proteins found on the surface of the target virus. This robust immune activation can lead to a higher incidence of AEs as the immune system reacts to the vaccine components.

Regarding the number of AEs and participant characteristics, there was a significant difference in the number of AEs in terms of gender, BMI category, and smoking status, with no significant difference noticed among other characteristics. A higher number of AEs was noticed among females compared to males which could also be related to gender disparity in coping with immune response, as discussed earlier. The association between the number of AEs and obesity status showed inconsistent results. There was no significant difference in the prevalence of AEs between any of the BMI categories after the first dose. However, more than half of the overweight (55.17%, n = 240) and 75% (n = 6) of the severely obese vaccinated individuals reported one to three AEs after the second dose (p = 0.03). Our finding is parallel to the controversy observed in the literature, as indicated in a very recent systematic review published in May 2023, which aimed to assess the safety and efficacy of COVID-19 vaccines specifically in individuals who were overweight or obese [28]. Among the included studies, 13 reported the use of BNT162b2 (Pfizer-BioNTech, USA), while four studies reported the use of ChAdOx-nCov19 (AstraZeneca, UK). In the mentioned systematic review, the available evidence did not provide conclusive indications regarding the overall safety of these vaccines in the overweight or obese population. In fact, the study concluded that there is a shortage of evidence to draw firm conclusions regarding the safety of the vaccine in this particular population. Therefore, it is imperative for healthcare professionals and policymakers to prioritize the monitoring of potential AEs of COVID-19 vaccinations in overweight or obese individuals.

Regarding smoking status, according to our study results, the percentage of asymptomatic vaccinated smokers versus non-smokers was 31.66% vs. 26.56% after the first dose and 55.28% vs. 45.25% after the second dose, respectively. In addition, the number of AEs among non-smokers was more than that for smokers after both the first and second doses but only significant after the second dose (p = 0.038). This lower incidence of AEs for COVID-19 vaccination among smokers was also observed nationally in one Iraqi study and in a study from Iran [18,26]. In fact, smoking has been shown to have immunosuppressive effects, potentially impairing the body’s ability to mount an optimal immune response. This can lead to reduced production of antibodies and potentially impact vaccine reactogenicity. A comprehensive systematic review was conducted to assess the impact of smoking on the humoral response to COVID-19 vaccines. Among the 23 studies examined, 17 reported significantly lower antibody titers or a more rapid decline in vaccine-induced IgG among current smokers compared to non-smokers [29], suggesting a detrimental effect of active smoking on the humoral response to COVID-19 vaccines. However, the precise pathophysiological mechanisms underlying this association have not been fully elucidated. More research is needed to better understand the specific mechanisms through which smoking may affect vaccine response and whether these effects translate into differences in AEs following vaccination.

Long-term adverse events

Our study followed the vaccinated individuals for long-term AEs up to one year after the second dose of vaccination. There were no major or chronic AEs with Pfizer and Sinopharm. However, among the 195 AstraZeneca-vaccinated individuals, there was only one long-term AE, i.e., myocarditis. The patient exhibited inappropriate exertional tachycardia and exertional dyspnea. Due to the persistence of these symptoms, the patient was referred to a cardiologist for evaluation, including echocardiography. The impression and diagnosis were established based on findings of subnormal left ventricular (LV) function and mild LV wall thickness increase. Obviously, viral infection is among the common causes of myocarditis. However, several vaccine types including COVID-19 vaccines can induce myocarditis as well [30]. Most case reports have observed that mRNA vaccines are a more common cause of myocarditis compared to other vaccines [31]. Still, few reports have documented myocarditis after vaccination with adenovirus vaccine, specifically AstraZeneca [32]. In fact, myocarditis after COVID-19 vaccination is more common in males and after the second dose which is typical in this case [31]. Vaccine-induced myocarditis is still an area of active research, and the specific mechanisms and etiology of this condition are not yet fully understood. However, the most possible explanation linked it to either direct viral infection, as some adenovirus-based vaccines, including AstraZeneca vaccine, use a modified adenovirus vector to deliver the genetic material of the target virus (in this case, the spike protein of SARS-CoV-2) into cells. It is possible that the adenovirus vector, or its replication, could directly infect cardiac cells, leading to inflammation and myocarditis [33]. The other possible explanation is related to immune response modulation through activating various components of the immune system by the vaccine effects on specific immune pathways or the release of certain cytokines and chemokines that may contribute to the development of myocarditis in susceptible individuals. The temporal relationship between the occurrence of myocarditis and the administration of the second dose of the vaccine, particularly when evident several weeks after vaccination, provides compelling evidence for a potential causal relationship that warrants special awareness.

Strengths and limitations

To our knowledge, this is the first study in Iraq with a prospective direct evaluation of AEs among the three vaccines with a relatively sufficient sample size. In addition, it is the first insight study in the Middle East (unless it is the first worldwide) to provide a one-year follow-up for the three vaccines to investigate their chronic AEs and long-term safety profiles. Another strength of our study is that the data collection was done through direct face-to-face interviews with professional healthcare staff which reduced information bias that can occur in other related web-based and retrospective studies. One limitation of this study is that it was conducted at the national level and did not include a comparison between countries globally. Another limitation is the potential for self-reporting bias in AE data collected from vaccinated individuals. However, we believe that this potential contamination would not have significantly impacted our results as we depended on well-trained assistant healthcare personnel for data collection under the direct and full supervision of the researchers with a double-checking process for data.

## Conclusions

The results from this study contribute to the growing body of evidence on COVID-19 vaccine safety. For all three vaccines, the majority of short-term AEs were mild and predictable while long-term follow-up revealed myocarditis associated with AstraZeneca. The most common short-term AEs reported in this study were fever, injection-site pain, myalgia, fatigue, and headache, which were more common in females, as observed in other studies as well. Remarkably, Pfizer was associated with the highest prevalence of AEs as opposed to Sinopharm. A significantly lower prevalence of AEs after the second dose across all three vaccines was observed. Understanding the patterns and characteristics of AEs is crucial for optimizing vaccine monitoring and improving public health strategies. By continuously evaluating and refining vaccine safety profiles, we can enhance public confidence and ensure the ongoing success of COVID-19 vaccination campaigns worldwide.

## Appendices

**Table 5:** Data collection form.

ID No	Code:
Name	
Agree to participate in the study	□ Yes □ No
Consent form has been explained to you	□ Yes □ No
Form completion date (dd/mm/yyyy)	
Phone number	
Email address	

Sex		□ Male □ Female
Date of birth (dd/mm/yyyy) and Age (years)		
Nationality		
Race		
City		
Residency		
Vaccination center name		
Level of education		□ None □ Primary □ Secondary □ University □ Postgraduate level (specify)
Occupation		
Height:	Weight:	BMI:
Economic status (income)		<500$ 500-1000$ 1000-3000$ 3000$ <
Blood group		□ A □ B □O □ AB
Do you have a chronic condition? ☐ Yes ☐ No ☐		DM, CVD, HT, CKD. B asthma, obesity, etc. If others specify:
Smoking status: ☐ Yes ☐ No ☐ Previously		□ I’ve never smoked □ I stopped smoking more than one year ago □ Yes, I currently smoke
Alcohol: ☐ Yes ☐ No ☐ Previously		
Hookah		□ Yes □ No
Are you currently pregnant? □ Yes □ No		Trimester: First □ Second □ Third □ Unknown
Are you taking regularly any medication(s)?		□ Yes □ No
If yes specify:		e) Corticosteroids
a) Statins □ Yes □ No □ Unknown		f) Anti-rheumatic (Immunosuppressive drugs)
b) Angiotensin-converting enzyme inhibitors		g) Immunosuppressive drugs
c) Angiotensin II receptor blockers		h) Antithrombotic/ platelet aggregation inhibitors
d) Non-steroidal anti-inflammatory drugs		i) Others
Specify:		

Date of First dose vaccination	Date of 2^nd^ dose vaccination
Product name	
Type of COVID-19 vaccine:	Pfizer; AstraZeneca; Sinopharm; others specify
Patch No	Patch No
Vaccination Card NO	Vaccination Card NO
Signs and symptoms after vaccination Fever Fatigue Headache Bodyache Chill Nausea Cough Loss of taste and smell shortness of breath	Others: specify fever > 38
Severity of side effects	Mild Moderate Severe
How long did it last?	Few days Several days
Have you received an influenza vaccine in the current influenza season (since October 2020)	□ Yes □ No □ Unknown
Were you infected before the vaccine? If Yes how long before the vaccine?	□ Yes □ No
Since the beginning of the pandemic in January 2020, have you tested positive for SARS-CoV-2?	□ Yes □ No
If yes, which test was used?	□ Antigen □ PCR □ Serology □ I don’t remember
IgG test result 21 days after the first dose	
IgG test result 14 days after the seconddose	

Post vaccine COVID-19 Infection	□ Yes □ No
Duration (after how many months)	1 month 2 months 3 months >3 months
If the first month, how many days after the vaccination?	
• Symptoms	
• Severity of symptoms	Mild Moderate Severe Critical
• Date of onset of symptoms	
• Date of PCR testing and PCR results	
• Clinical course of illness (including outpatient and inpatient visits)	
